# Multimodal Biosensing on Paper-Based Platform Fabricated by Plasmonic Calligraphy Using Gold Nanobypiramids Ink

**DOI:** 10.3389/fchem.2019.00055

**Published:** 2019-02-08

**Authors:** Andreea Campu, Laurentiu Susu, Filip Orzan, Dana Maniu, Ana Maria Craciun, Adriana Vulpoi, Lucian Roiban, Monica Focsan, Simion Astilean

**Affiliations:** ^1^Nanobiophotonics and Laser Microspectroscopy Center, Interdisciplinary Research Institute on Bio-Nano-Sciences, Babes-Bolyai University, Cluj-Napoca, Romania; ^2^Biomolecular Physics Department, Faculty of Physics, Babes-Bolyai University, Cluj-Napoca, Romania; ^3^Nanostructured Materials and Bio-Nano-Interfaces Center, Interdisciplinary Research Institute on Bio-Nano-Sciences, Babes-Bolyai University, Cluj-Napoca, Romania; ^4^Univ Lyon, INSA-Lyon, Université Claude Bernard Lyon 1, MATEIS, UMR, CNRS, Villeurbanne, France

**Keywords:** biodetection, plasmonic paper, nanoplatform, LSPR, SERS, MEF

## Abstract

In this work, we design new plasmonic paper-based nanoplatforms with interesting capabilities in terms of sensitivity, efficiency, and reproducibility for promoting multimodal biodetection via Localized Surface Plasmon Resonance (LSPR), Surface Enhanced Raman Spectroscopy (SERS), and Metal Enhanced Fluorescence (MEF). To succeed, we exploit the unique optical properties of gold nanobipyramids (AuBPs) deposited onto the cellulose fibers via plasmonic calligraphy using a commercial pen. The first step of the biosensing protocol was to precisely graft the previously chemically-formed p-aminothiophenol@Biotin system, as active recognition element for target streptavidin detection, onto the plasmonic nanoplatform. The specific capture of the target protein was successfully demonstrated using three complementary sensing techniques. As a result, while the LSPR based sensing capabilities of the nanoplatform were proved by successive 13–18 nm red shifts of the longitudinal LSPR associated with the change of the surface RI after each step. By employing the ultrasensitive SERS technique, we were able to indirectly confirm the molecular identification of the biotin-streptavidin interaction due to the protein fingerprint bands assigned to amide I, amide III, and Trp vibrations. Additionally, the formed biotin-streptavidin complex acted as a spacer to ensure an optimal distance between the AuBP surface and the Alexa 680 fluorophore for achieving a 2-fold fluorescence emission enhancement of streptavidin@Alexa 680 on the biotinylated nanoplatform compared to the same complex on bare paper (near the plasmonic lines), implementing thus a novel MEF sensing nanoplatform. Finally, by integrating multiple LSPR, SERS, and MEF nanosensors with multiplex capability into a single flexible and portable plasmonic nanoplatform, we could overcome important limits in the field of portable point-of-care diagnostics.

## Introduction

The design of innovative biosensing devices proving portability, high efficiency, and sensitivity, ease of use and low-cost increases continuously the research interest focused on the development of bio-nanotechnologies for specific diagnostic applications (Jiang et al., 2017). Such promising point-of-care (POC) devices, able to ensure the premature diagnosis of high-risk diseases by specifically detecting the corresponding biomarker in view of early and appropriate treatment, can be plasmonic paper-based nanoplatforms due to their advantages like facile and rapid fabrication, high specific surface area, flexibility, or excellent wicking ability due to capillary forces (Kim et al., 2018). Gold nanostructures of different shapes (Li S. et al., 2017) or compositions (Zhang et al., 2014; Zhang L. et al., 2018) organized in various assemblies (Si et al., 2014) are on their own promising for a large variety of biomedical applications, all aiming to ease the way for rapid diagnostic procedures. Nevertheless, their immobilization on suitable substrates, such as paper, expands the list of advantages and subsequently their applicability field. Additionally, the robustness and the versatility of such paper-based POC plasmonic chips have been proven to be an effective way to overcome the limitations of commercially available detection kits, enabling a specific biodetection of an ultra-low target analyte concentration using reduced sample volume via plasmonic readout (Lee et al., 2011).

Furthermore, by the agile manipulation of the synthesized plasmonic nanoparticle' shape and size, and implicitly, of their generated localized surface plasmon resonance (LSPR) response (Stewart et al., 2008), they can be transferred onto flexible paper cellulose substrates to implement LSPR biosensing paper-based nanoplatforms. It is well-known that longer LSPR peak wavelengths generate increased bulk refractive index sensitivities (RIS) that subsequently will improve the detection limit of the analyte of interest (Rye et al., 2018). Gold bipyramids (AuBPs), as attractive anisotropic nanotransducers, respond well to this demand, offering the possibility to be synthesized in such way that will induce a fine tailoring of the longitudinal LSPR band over the entire electromagnetic spectrum. Our previous study demonstrates the feasibility of these diamond-like shaped nanostructures to be implemented as highly sensitive LSPR immunosensors in aqueous solution (Campu et al., 2018). Briefly, the ability of different aspect ratio AuBPs to efficiently detect target molecules was proven for antigen-antibody interactions both simple “proof-of-concept” reactions, such as the biotin-streptavidin recognition, and more complex real-sample reactions like anti-human IgG–human IgG binding. Concretely, the fine tuning of the LSPR response allows the optimization of the biosensing colloidal design so far as reaching the best experiment-suited LSPR sensitivity. Additionally, it has been recently proven, both experimentally and theoretically, that these sharp nanoparticles exhibit a higher RIS, compared to Au nanorods, for example as well as better figure of merit (FOM) values, narrow FWHM and huge local field enhancement at their two sharp tips (Li et al., 2015; Rao et al., 2015; Zhu et al., 2016). In fact, their sharp features provide the basis for surface enhanced Raman spectroscopy (SERS), making the AuBPs promising tools for ultrasensitive biosensing applications (Reguera et al., 2017). In this context, AuBPs reveal highly appealing advantages which offer the opportunity to not only develop LSPR-based paper nanoplatforms but also to implement them as three-dimensional (3D) SERS based paper-nanoplatforms, by profiting from the micro/nanoscale structure of paper, which due to its cellulose fibrous strands and smaller microfibers interwoven together, is able to absorb, and drive the plasmonic nanoparticles like a natural microfluidic channel (Liana et al., 2012). More exactly, the cellulose fibers can secure a highly efficient volumetric SERS ultra-detection *via* the generation of the so-called intrinsic plasmonic hotspots in paper triggered by the enhanced local electromagnetic field (Ngo et al., 2013; Tian et al., 2016; Dalla Marta et al., 2017; Oliveira et al., 2017; Ashley et al., 2018; Zhang S. et al., 2018). Interestingly, combined micro and nanofibers of cellulose could also allow a 3D ultrasensitive detection of the biomarkers of interest *via* metal-enhanced fluorescence (MEF) through labeling them with fluorophores and nanostructures, thus improving the detection capability of the designed paper-based plasmonic nanosensor. The development of MEF biosensing nanoplatforms can be challenging since MEF is completely dependent on the distance between the metal surface and the fluorophore, which is usually obtained by employing a spacer with a length ranging from 5 to 20 nm. If the necessary distance is not acquired, the quenching phenomenon occurs decreasing the fluorescence intensity of the fluorophore (Geddes and Lakowicz, 2002). Therefore, the careful selection of the spacer and gaining control over the labeling process is vital for the fabrication of MEF nanosensors. Such MEF point-of-care devices were demonstrated to be effective for lowering the detection limit of common detection assays such as immunofluorescence assay (Nooney et al., 2010; Liu et al., 2018) or for the successful ultra-low detection of cancer biomarkers (Park et al., 2018; Della Ventura et al., 2019). In this optical process, where fluorophores should be placed near the metal surface at a certain distance to generate fluorescence emission amplification (Pompa et al., 2006; Deng and Goldys, 2012), AuBPs can act as intriguing MEF nanoantennas through giving arise at their two sharp tips to a significantly enhanced local field, which can be further amplified due to the promising structure of the paper by allowing the formation of the intrinsic plasmonic hotspots. Theoretically, if the LSPR band presents a substantial overlap with the excitation spectrum of the fluorophore, the LSPR of the nanoparticles is able to enhance the absorption of the incident light employed for photoexcitation and consequently the energy will be transferred to the fluorophore (Aslan et al., 2005). On the other hand, active MEF nanoplatforms with potential to enhance the near infrared (NIR) emission are highly desirable thus opening interesting routes for innovative diagnostic devices not explored so far. Nevertheless, currently, a strong scientific priority is the efficient integration of multiple nanosensors proving multiplexing capabilities within inexpensive easy-to-use portable paper-based sensing nanoplatforms in order to obtain a miniaturized detection system with improved control and sensitivity for further clinical diagnostics applications. Surprisingly, innovative plasmonic paper-based nanoplatforms with highly controllable broad-range tunability of the LSPR response, especially in the NIR biological windows, enabling a confident enhanced multimodal plasmonic SERS and MEF detection of specific antigen-antibody recognition interactions are still lacking from the literature.

Therefore, in this work, we answer the above-mentioned demands by developing a new concept of nanosensor directly on paper, using a commercial pen filled with plasmonic AuBPs as plasmonic ink, to test the detection of the specific biotin-streptavidin recognition interaction as a “proof-of-concept.” To note that, the immersion approach excludes—in general—spatial multiplexing, hindering the design of spatially isolated and chemically selective test surfaces and limiting the further biodetection to only one specific target analyte (Tian et al., 2014). Using this simple nanoplasmonic calligraphy approach, we are able to control and tune the plasmonic properties of the nanoplatforms by drawing separate chemically active plasmonic line domains on the same substrate comprising AuBPs with three different aspect ratios (herein, AuBP 626, AuBP 713, AuBP 860). The employed method does not only present economical and time-related advantages being inexpensive, easy to implement and rapid, but also shows a high efficiency in terms of nanoparticle deposition due to the well-preserved optical properties of the colloidal AuBPs after the immobilization, the as-prepared plasmonic nanoplatform maintaining a high LSPR sensitivity and SERS activity. The proposed biosensing protocol involves the prior chemical preparation of the biotin label with the p-aminothiophenol (p-ATP) molecule (noted further as p-ATP@Biotin), which acts simultaneously as: (i) the capture element for the target streptavidin detection; (ii) the linker to the Au surface which covalently binds to the thiol group of the p-ATP molecule, (iii) the Raman label due the p-ATP Raman reporter, and (iv) the spacer needed for MEF to occur, ensuring an optimal metal-to-fluorophore distance necessary to achieve an increase in the florescence intensity from Alexa680 dye conjugated with streptavidin. Subsequently, a monolayer of streptavidin conjugated Alexa680 complex (noted further as streptavidin@Alexa680) was grafted on the previously biotinylated plasmonic paper nanoplatforms for the recognition interaction to be validated. The successful specific capture of the streptavidin molecule due to the strong antigen-antibody interaction was demonstrated and evaluated using three complementary analysis techniques. As a result, a red-shift of the extinction spectrum recorded after each immobilization step confirms the LSPR sensing capability of the designed nanoplatform. By the monitorization of the p-ATP vibrational modes along with the amplification of the protein residues characteristic Raman bands, the identification of the molecules involved in the process was successfully achieved by SERS detection. Moreover, epifluorescence measurements indicate the enhancement of the Alexa680 emission, thus proving the MEF sensing abilities of the as-designed nanoplatform. In conclusion, such engineered paper-based plasmonic platforms represent a real challenge with promising results for medical diagnostics, allowing the development of innovative plasmonic multiplex paper-based POC biochips.

## Materials and Methods

### Chemicals

Tetrachloroauric acid (HAuCl_4_•4H_2_O, 99.99%), cetyltrimethylammonium bromide (CTAB, 96%), L-ascorbic acid (C_6_H_8_O_6_, 99%), citric acid (C_6_H_8_O_7_), cetyltrimethylammonium chloride (CTAC), hydroxyquinoline (C_9_H_7_NO, 99%), sodium borohydride (NaBH_4_, 99%), silver nitrate (AgNO_3_, 99%), p-aminothiophenol (p-ATP), Biotin N-hydroxysuccinimide ester (Biotin-NHS), Phosphate buffered saline (PBS, pH 7.2), and Whatman® qualitative filter paper, Grade 1 (Whatman no. 1) were purchased from Sigma-Aldrich. Invitrogen™ Alexa Fluor® 680 Streptavidin Conjugate was acquired from ThermoFischer Scientific. All chemicals were used without further purification. Ultrapure water (resistivity ~ 18.2 MΩ) was used as solvent throughout the experiments.

### Colloidal Gold Bipyramids Synthesis

Colloidal gold bipyramidal-shaped nanoparticles (AuBPs) were synthesized using a previously described seed-mediated growth approach (Navarro et al., 2012; Chateau et al., 2015). The Au seeds were prepared by mixing 1 M salt with an aqueous 25 w% CTAC solution, 0.25 M HNO_3_, 50 mM NaBH_4_ as reducing agent and 1 M citric acid under vigorous stirring. The final mixture was thermally treated at 80°C for 60–90 min. The anisotropic nanoparticles were grown in the presence of 25 mM HAuCl_4_, 45 mM CTAB stabilizing agent solution, 5 mM AgNO_3_, and of 0.4 M HQL as reducing agent using seed volumes of 300, 65, and 12 μl for AuBPs with longitudinal LSPR at 626, 713, and 860 nm, respectively. The solutions were left at 45°C for 50 min. In view of further use, two purification steps were employed by centrifugation at 8,000 rpm for 15 min and redispersion in ultra-pure water.

### Fabrication of Paper-Based Plasmonic Nanoplatforms Through Plasmonic Calligraphy

A commercial Schneider pen, with an empty refillable cartridge, was purchased from a local office supply store. Concentrated colloidal AuBPs, as plasmonic inks, were used to draw spatially isolated lines, with a width of ~1 mm on Whatman no. 1 paper strips. The paper-based substrates with different plasmonic lines were left to dry at room temperature. The protocol was repeated, by drawing supplementary lines in the same position, in order to increase the density of the AuBPs, until the as-designed paper-based plasmonic nanoplatform was optimized for further biosensing use.

### Recognition Element Labeling and Biosensing Protocol

Prior to the implementation of the biosensing protocol, the biotin molecule was chemically labeled with the Raman reporter p-Aminothiophenol (p-ATP) using a previously described approach (Jiang et al., 2009; Campu et al., 2018). Briefly, under controlled chemical conditions, p-ATP (36 mg, 0.318 mmol) was added dropwise to a commercially available biotin-NHS solution (100 mg, 0.318 mmol). The mixture was left undisturbed overnight and following an evaporation and precipitation treatment, the final product (referred as p-ATP@Biotin) was dissolved into methanol for further use. The stock solution concentration was determined to be 15 mM.

**Scheme 1:**
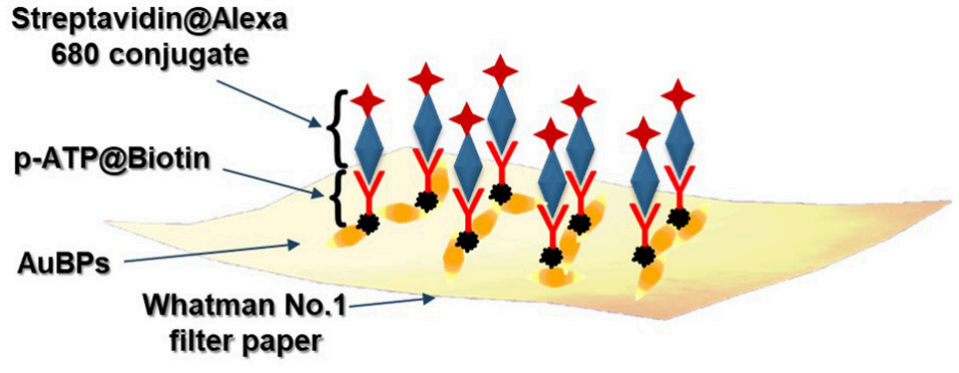
Schematic illustration of the proposed biosensing protocol.

The proposed biosensing protocol, schematically illustrated in Scheme 1, implies the dropwise successive addition of the p-ATP@Biotin (1.5 mM) and Streptavidin@Alexa 680 conjugate (0.25 mg/ml, in PBS pH 7.2) onto the as-prepared plasmonic lines of the paper-based substrates. To note that the non-specific binding was avoided using 1% BSA in PBS solution. In order to evaluate the LSPR limit of detection, different target complex (herein Streptavidin@Alexa 680 conjugate) concentrations were tested (from 0.01 to 1 mg/mL).

### Characterization Methods

The extinction spectra of the colloidal AuBPs were recorded using a Jasco V-670 UV-Vis-NIR spectrophotometer with a 2 nm bandwidth and 1 nm spectral resolution. The size and morphology of the synthesized AuBPs in aqueous solution having different aspect ratios were examined using a FEI Tecnai F20 field emission Transmission Electron Microscope (TEM), operating at an accelerating voltage of 200 kV and equipped with Eagle 4 k CCD camera. The colloid was added dropwise onto a carbon film covered copper grid for TEM analyses. Dynamic light scattering (DLS) and Zeta Potential measurements of the colloidal AuBPs were performed using a Nano ZS90 Zetasizer analyzer from Malvern Instruments equipped with a He-Ne laser (633 nm, 5 mW). The used analysis parameters were a scattering angle of 90° and temperature of 25°C. All samples were measured three times and the mean value has been reported.

After the controlled immobilization of the AuBPs on Whatman paper, the plasmonic responses of the new as-formed paper-based nanoplatforms were collected using a portable Ocean Optics USB 4,000 optical UV-Vis spectrophotometer coupled to a ZEISS Axio Observer Z1 inverted microscope with 10x ZEISS objective (NA = 0.45) through an optical fiber with a core diameter of 600 μm. The extinction spectra were recorded in absorption mode, using 5 accumulations and 50 ms integration time, the spectral resolution of the spectrophotometer being 0.2 nm. Subsequently, the morphology and the uniformity of the new nanoplatforms were investigated by Scanning Electron Microscopy (SEM) using a FEI Quanta 3D FEG dual beam scanning electron microscope operating at an accelerating voltage of 30 kV. The plasmonic nanoplatforms were sputtered using a Q150R ES automatic Sputter Coater, in an argon atmosphere, with 5 nm gold layer for 10 min prior to the SEM investigation in order to inhibit charging, reduce thermal damage, and improve the secondary electron signal required for topographic examination in the SEM. High Resolution TEM (HRTEM) images were then recorded using a Jeol 2010F electron microscope working at 200 kV. For HRTEM observation, the plasmonic nanoplatforms were wetted with alcohol, then scratched with a scalpel and the debris was suspended in alcohol using ultra-sonication for 15 min to disperse it. A droplet of the solution was dribbled on a holey carbon grid 300 mesh microscopy grid and let dry.

The SERS spectra were recorded with a portable Raman Systems R3000CN spectrometer, equipped with a 785 nm diode laser coupled to a 100 μm optical fiber. The laser measurements were acquired at a laser power of 170 mW and 30 s per spectrum integration time.

Fluorescence emission measurements were collected at room temperature using an epifluorescence accessory (EFA 383 module) for Jasco LP-6500 spectrofluorometer with a 1 nm spectral resolution, equipped with a Xenon lamp as excitation source, using excitation and emission bandwidths of 3 nm. Fluorescence spectra were recorded in the wavelength range of 660–900 nm employing a fixed excitation wavelength at 660 nm.

## Results and Discussion

### Design of New Paper-Based Sensing Nanoplatforms Through Plasmonic Calligraphy

In view of the further implementation as plasmonic inks to fabricate paper-based plasmonic nanoplatforms, the as-synthesized AuBPs in aqueous solution were thoroughly characterized both optically and morphologically. As the seed volume in the synthesis process is decreased, the AuBPs exhibit an increase in size -as expected- and therefore an increase of the longitudinal LSPR wavelength, allowing the fine tunability of the gold diamond-like particles spanning for the visible to the near-infrared regions of the electromagnetic spectrum. Supplementary Figure 1A represents the normalized extinction responses of three synthesized colloidal solutions with different aspect ratios, the two characteristic surface plasmonic resonances of the AuBPs are clearly distinguished (Liu and Guyot-Sionnest, 2005), showing a stationary transversal contribution at around 510 nm and a longitudinal LSPR band with spectral positions at 626 (black spectrum), 713 (blue spectrum), and 860 nm (green spectrum), respectively. Moreover, corresponding TEM microscopic images (Supplementary Figure 1B) indicate AuBPs with well-defined shape and high monodispersity for each of the three selected samples. Using a commercially available analysis program, ImageJ toolkit, the dimensions of the AuBPs have been determined by measuring the length and width of at least 100 nanostructures (Supplementary Table 1). The assessed sizes were than compared with the obtained Dynamic Light Scattering (DLS) results, the hydrodynamic diameter data being in good agreement with the size analysis from the TEM investigation. Furthermore, Zeta Potential measurements appraise a positive 27 ± 0.3 mV surface potential (Supplementary Table 1), indicating a high stability, and more importantly, their possibility to be further immobilized on the negatively charged cellulose fibers via electrostatic interaction.

**Figure 1:**
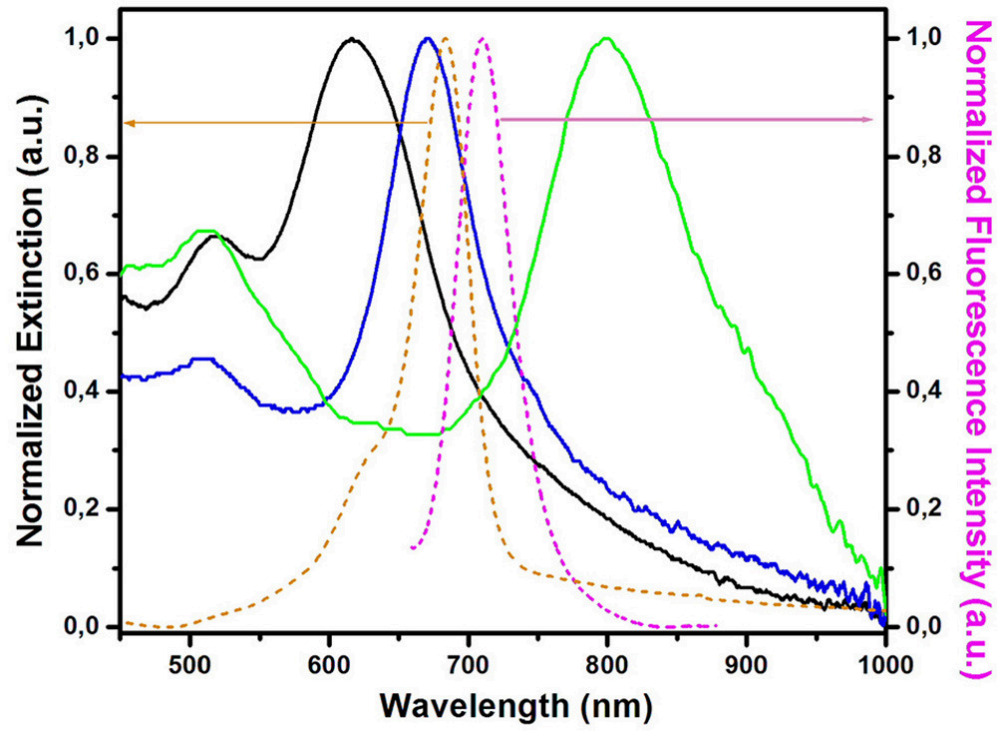
Spectral overlap between the normalized extinction spectra of the plasmonic nanoplatforms (solid spectra) after the AuBPs immobilization (paper@AuBPs 618–black spectrum, paper@AuBPs 675–blue spectrum, and paper@AuBPs 800–green spectrum) together with the absorption (orange dashed spectrum), and fluorescence emission (magenta dashed spectrum) of the free streptavidin@Alexa 680 conjugate adsorbed on the bare Whatman paper.

For the fabrication of the tunable plasmonic paper-based nanoplatforms, the colloidal AuBPs were used to fill empty cartridges of commercially available ballpoint pens, performing thus as colloidal plasmonic inks. Common Whatman No. 1 laboratory filter paper was used as substrate for the immobilization of the AuBPs due to its 3D structure composed of α-cellulose (98%), micro scale (~10 μm) cellulose fibrous strands and smaller microfibers (~0.4 μm) with nanofibers interwoven together (Hankus et al., 2012). The simple and low-cost fabrication protocol was employed for the three colloidal plasmonic solutions, allowing to draw spatially isolated plasmonic lines on Whatman paper strips. Nevertheless, the proposed method is rapid compared to the time-consuming printing technique. Subsequently, the next step was to assess the LSPR responses of the fabricated plasmonic nanoplatforms, and, as such, the extinction spectra were recorded after the immobilization of the AuBPs onto the paper substrates (Figure 1) and compared with the colloidal optical responses (Supplementary Figure 1A). As expected, we recorded blue-shifts of the longitudinal LSPR bands of 13–63 nm while the transversal band maintains its position, as a consequence of the decrease of the refractive index from 1.333 (water) to 1.0003 (air) (Mayer and Hafner, 2011), obtaining thus tunable plasmonic paper-based nanoplatforms with LSPR responses at 618 nm (Figure 1–black spectrum) (further referred as paper@AuBPs 618), 675 nm (Figure 1–blue spectrum) (further referred as paper@AuBPs 675), and 800 nm (Figure 1–green spectrum) (further referred as paper@AuBPs 800), respectively.

Notably, apart from the recorded LSPR blueshift, the extinction spectra do not exhibit any changes, thus confirming the preserved optical plasmonic signatures of the colloidal AuBPs. To note that the recorded LSPR response after the AuBPs immobilization onto the paper is stable in time, yet several washing steps did not induce any alterations of the recorded LSPR response even after 4 months hence proving high stability of the LSPR response (Supplementary Figure 2). It is worth mentioning that compared to our previous study, where were used the AuBPs in aqueous solution for the biosensing applications (Campu et al., 2018), in the current work, the nanoparticles were intentionally synthesized to generate after the immobilization onto the paper fibers longitudinal LSPR bands located in and out of resonance with the emission maximum of the fluorophore conjugate, herein streptavidin@Alexa 680, chosen further in the sensing application as target analyte, in order to have a better understanding of the nanoplatforms behavior as possible MEF biosensing devices. As such, Figure 1 shows the spectral overlapping between the extinction spectra of the new designed plasmonic lines and the absorption (orange dashed spectrum), and fluorescence emission (magenta dashed spectrum) of the free streptavidin@Alexa 680 conjugate adsorbed on the bare Whatman paper.

**Figure 2:**
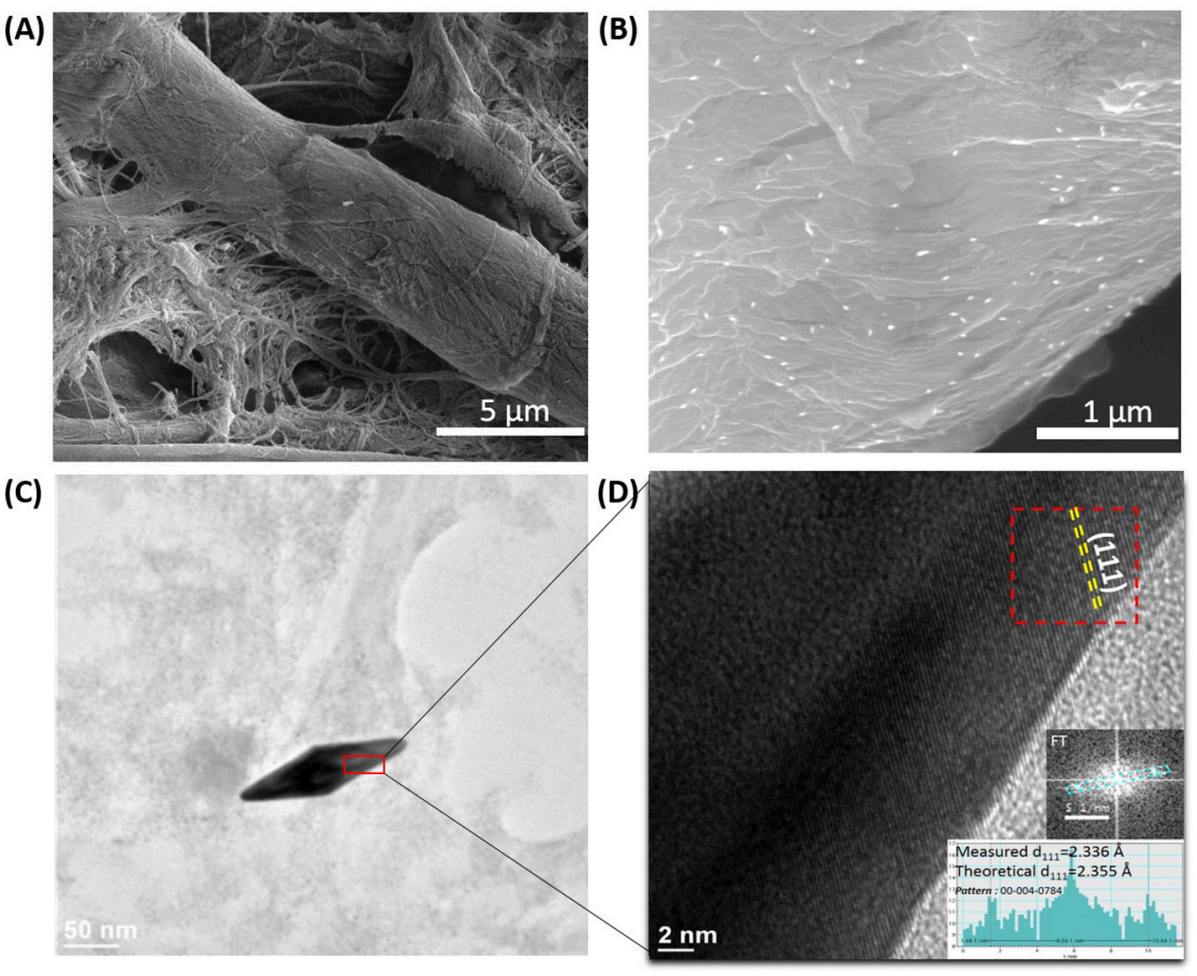
Representative SEM images of the bare Whatman paper **(A)** and the selected paper@AuBPs800 calligraphed nanoplatform **(B)**; HRTEM microscopic image of a single AuBP at high resolution **(C)**; HRTEM image showing the (111) family planes faces of the AuBPs. Inset: the Fourier transform made on the marked region and its measurements **(D)**.

Subsequently, the morphology of the plasmonic calligraphed substrates was evaluated in order to determine the way the nanoparticles are immobilized on the fibrous strands of the cellulose. In this context, just for exemplification, representative SEM images of the paper substrate were successfully recorded before (Figure 2A) and after (Figure 2B) one-line plasmonic calligraphy using colloidal AuBPs 860 as ink. In particular, Figure 2B illustrates an individual distribution of the AuBPs onto the paper fibers without large-scale aggregation. This observation is also supported and confirmed by the LSPR measurements, which exhibit the preserved optical response of the AuBPs after the deposition process, again, without showing significant contributions from aggregation processes. For a better visualization, the HRTEM technique was employed thus confirming the successful immobilization of the nanostructures. To note that the AuBPs have not been damaged in any way during the deposition process, HRTEM images (Figure 2C) show the identical shape and size as prior the immobilization.

Moreover, the AuBPs are exposing the (111) family planes at the surface (Figure 2D). In fact, the attachment of the AuBPs onto the paper fibrous strands relies on the electrostatic interaction between the negatively charged cellulose fibers having hydroxyl groups on its surface (Azizi Samir et al., 2005; Li J. et al., 2017) and the positively charged CTAB bilayer at the AuBPs surface. The binding involves the faces of the nanostructure, leaving the tips of the AuBPs exposed and easily accessible to analyte molecules.

### Controlling the Optical Density and Bulk Refractive Index Sensitivity

**Figure 3:**
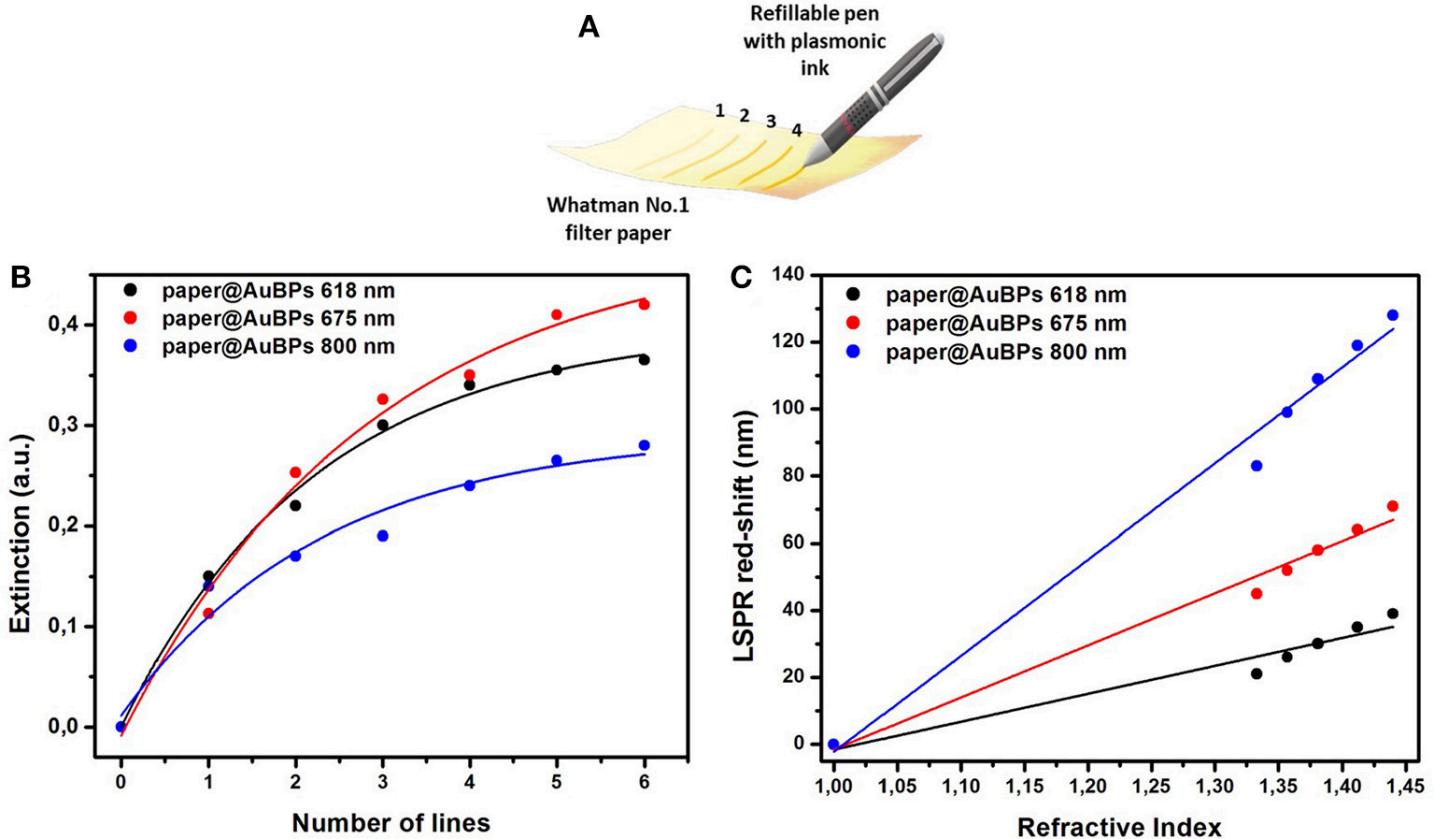
Schematic illustration of the drawing process employing commercial pen filled with AuBPs as colloidal ink in order to calligraph different numbers of re-traced plasmonic lines **(A)**; Dependence of the extinction intensity of the longitudinal LSPR band of the previously designed paper@AuBPs 618–black plot, paper@AuBPs 675–red plot, and paper@AuBPs 800–blue plot, as function of number of plasmonic lines traced **(B)**; Plots depicting the linear dependence of the longitudinal LSPR position on the bulk refractive index for the tunable plasmonic paper based nanoplatforms, i.e., paper@AuBPs 618, paper@AuBPs 675, paper@AuBPs 800, respectively **(C)**.

In order to enhance the optical efficiency of our nanoplatform for the future biosensing applications, controlling the optical density of the substrates is highly desirable. The main challenge is to increase the density up to a maximum, in such a manner that the plasmonic nanoparticles are captured and stabilized on the 3D cellulose microfibers network by a random arrangement which permits the interrogation of both transversal and longitudinal plasmonic modes. In this context, the proposed plasmonic calligraphy approach, schematically illustrated in Figure 3A, is well-suited for the task. The controlled repetition of the protocol by re-tracing new lines with plasmonic ink over previously drawn and dried lines ensures the manipulation of the optical density on paper. Therefore, plasmonic paper-based substrates with increasing number of lines were prepared (Supplementary Figure 3) and their UV-Vis spectra was collected in order to monitor the extinction intensity of the longitudinal LSPR band, which was then plotted as a function of the number of drawn plasmonic lines (Figure 3B).

The optical density saturates at a repetition rate of 5–6 for all plasmonic nanoplatforms concluding that we can easily control the optical density of the paper-based substrates and indirectly that we can load the cellulose fibers with a higher AuBPs concentration leading to improved detection abilities. To note that the extinction spectra were collected on each plasmonic line from a minimum of 4 different regions and the relative standard variation of the optical density was found to be around 6% (data not shown).

The biosensing capabilities were evaluated by assessing the *bulk* LSPR sensitivity of the as-prepared paper-based nanoplatforms having tunable plasmonic responses. In this matter, water-glycerol mixtures with different glycerol concentrations (0, 20, 40, 60, and 80%) and calculated refractive index (RI) ranging from 1.333 (water) to 1.473 (80% glycerol) were dropwise added on the plasmonic lines with saturated optical density in order to induce a refractive index change in the nanoparticle environment. As the RI is increased, the longitudinal LSPR band red-shifts for each plasmonic nanoplatform, and by subsequently plotting the recorded LSPR red-shift as a function of the RI, the bulk sensitivity could be determined from the linear fit. As a result, Figure 3C shows the linear regression obtained for each designed plasmonic paper nanoplatform, and from their slopes, bulk refractive index sensitivities (RIS) with values from 83 to 287 nm/RIU were established. The as-obtained values are comparable with the RIS values in solution (Campu et al., 2018) concluding in a high sensitivity for biodetection applications.

### Multimodal LSPR-SERS-MEF Biosensing

As previously stated, enhanced biosensing is an important route to detect low concentration of biomarkers of interest, and therefore, the design of a multimodal detection platform still represents a major key challenge that should be addressed. In this matter, it is undoubtedly challenging to design such miniaturized and portable sensing platforms on the same substrate—as innovative immunosensor—that allows the specific detection of the antigen-antibody recognition binding. To date some of the current methods used for protein detection are for example Enzyme-Linked Immunosorbent Assay (ELISA), Immuno-fluorescence and Polymerase Chain Reaction (PCR). These analysis techniques are commonly used in the medical field, but they have some limitations, which we try to overcome with the proposed plasmonic paper-based nanoplatform. For instance, ELISA is a popular method, the preparation and the analysis of the test is time-consuming and complicated. Even though it is able to detect nanomolar concentrations, it relies only on colorimetric techniques and below 10^−6^ M the quantification of the analyte concentration is difficult due to reproducibility issues. Similar to ELISA are PCR and immunofluorescence tests which have sensitivities close to ELISA but are more complex and can provide false positive responses. Moreover, these procedures are complex and tedious involving a high number of steps and need to be performed in a professional laboratory under special conditions. Surprisingly, despite the implementation of different plasmonic transducers strategies in literature to highlight antigen-antibody binding events resulting from different immunological reactions, MEF in NIR for fluorescent biosensing hasn't been yet successfully employed with SERS and LSPR for improving their applicability in biodetection. As such, by exploiting the optical tunability of AuBPs, and implicitly the electromagnetic intensity of their localized hot-spots, together with a correct choice of selected fluorophore as well as of the optimal distance between fluorophore and AuBPs surface, it should be possible to engineer such multimodal detection platforms.

**Figure 4:**
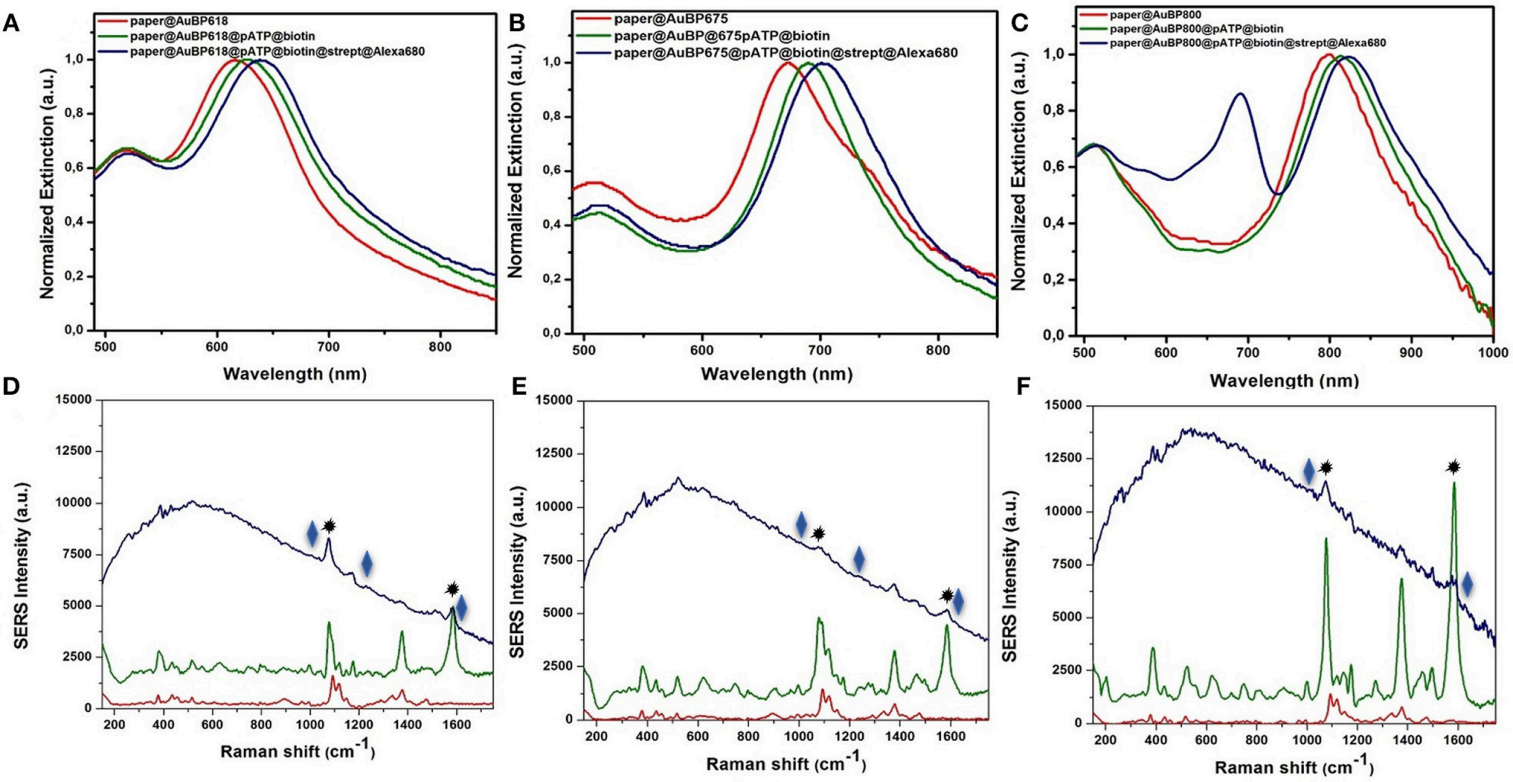
Normalized extinction spectra **(A–C)** together with the recorded SERS spectra **(D–F)** of the as-designed paper-based plasmonic nanoplatforms with tunable plasmonic response at paper@AuBP 618, paper@AuBP 675, and paper@AuBP 800, respectively (red spectra), after their biotinylating process (green spectra), and after the specific capture of target streptavidin (blue spectra), proving the capability of our nanoplatforms for dual LSPR-SERS detection. Excitation: 785 nm laser line.

In this context, the feasibility of the above-designed and optimized three plasmonic lines calligraphed on the same paper platform with different AuBPs inks to detect the biotin-streptavidin interaction as a “proof of concept” is subsequently investigated using a recently reported and adapted biosensing protocol (Campu et al., 2018) schematically illustrated in Scheme 1. An important change has been made to the reported biosensing protocol by choosing as target molecule the streptavidin@Alexa 680 conjugate thus adding a fluorophore placed due to the p-ATP@biotin@streptavidin spacer at a convenient distance from the Au surface and, implicitly, extending the detection capabilities of the developed nanosensors. Briefly, as recognition element for the specific detection of the target streptavidin, we covalently linked the p-ATP Raman reporter with Biotin-NHS in order to produce an active biosystem that is able to bind to the exposed ends of the immobilized AuBPs on paper through strong Au-S interactions (Zong et al., 2011), obtaining an active biotinylated plasmonic paper nanoplatform. The successful activation step with this previously formed pATP@Biotin system was first proved by the recorded extinction spectra on three traced plasmonic lines before (Figures 4A–C–red spectra) and after the p-ATP@Biotin immobilization (Figures 4A–C–green spectra). All the plasmonic nanoplatforms exhibit a red-shift of the longitudinal LSPR band between 13 and 17 nm, attributed to the change of the surface RI, as presented in Figures 4A–C–green spectra. But, for the specific identification of the p-ATP@Biotin, SERS measurements are employed secondly since it is a more sensitive tool for the analysis of the Raman fingerprint associated with the molecule of interest. Concretely, Figures 4D–F–green spectra present comparative SERS spectra of all the biotinylated plasmonic lines having different LSPR responses using a 785 nm excitation laser wavelength from a miniportable Raman spectrometer. As expected, the successfully grafting of the p-ATP@Biotin onto the paper-based plasmonic lines is proved by the presence of the characteristic vibrational modes of the p-ATP molecules located at 391 cm^−1^, 1,079 cm^−1^ (C-S stretching), 1,176 cm^−1^, 1,585 cm^−1^ (C-C stretching), but also the specific Raman bands of the biotin structures are located at 746 cm^−1^ (CN_2_ wagging), 1,043 cm^−1^ (C-C stretching valeric acid chain), 1,236 cm^−1^ (C-N stretching + N-H bending), and 1,456 cm^−1^ (CH_2_ rocking) (Galarreta et al., 2011; Campu et al., 2018).

To note that, the Raman spectrum of the bare Whatman paper itself (Supplementary Figure 4–black spectrum) and paper@AuBPs with different optical responses (Figures 4D–F and Supplementary Figure 4–red spectra) doesn't overlap with the p-ATP bands (Figures 4D–F–green spectra). On the other hand, we observe that all SERS lines are active, revealing characteristic bands of p-ATP@Biotin complex, but their SERS efficiencies are different, the highest SERS effect occurs for the paper@AuBP800 under excitation wavelength at 785 nm. It should mention that all the SERS lines were exposed to the same p-ATP@Biotin concentration.

Subsequently, the capture of the target streptavidin protein by the biotinylated paper-based plasmonic nanoplatforms leads to an additional red-shift between 14 and 18 nm (Figures 4A–C, blue spectra), confirming its specific detection and implicitly the successful biotin-streptavidin recognition interaction. To note, that the new absorption band at 680 nm observed in the Figure 4C–blue spectrum correspond to the absorption band of Alexa 680 fluorophore conjugated with streptavidin, in the other two cases (Figures 4A,B–blue spectra), this band overlaps with the longitudinal LSPR bands. The LSPR sensitivity was experimentally determined for the selected paper@AuBP 675 by calculating the limit of detection. Concretely, we plotted the red-shifts of the longitudinal LSPR response as a function of the target streptavidin@Alexa 680 complex concentrations (Supplementary Figure 5). Notably, the corresponding SERS investigations of the biotinylated plasmonic lines after the specific capture of streptavidin@Alexa 680 complex (Figures 4D–F, blue spectra) clearly evidence, besides the dominant fluorescence signal from the Alexa 680 fluorophore and still the presence of the vibrational modes of p-ATP (1,079 and 1,585 cm^−1^, marked with the black star), the appearance of characteristic Raman bands of the detected streptavidin protein. In particular, the new bands at 1,630, 1,244, 1,010 cm^−1^ (marked with the blue rhombus) are assigned to amide I, amide III, and Trp vibrations of streptavidin, confirming thus the successful biotin-streptavidin recognition interaction and the possibility of such calligraphic SERS lines to be further implemented in other relevant antigen-antibody interactions. Furthermore, in the case of paper@AuBP 800 platform we observe a higher fluorescence emission signal compared to the other two nanoplatforms due to the resonant excitation of the 800 nm longitudinal LSPR band due to the use of the 785 nm laser line, proving the possibility to detect SERS and MEF signals together.

**Figure 5:**
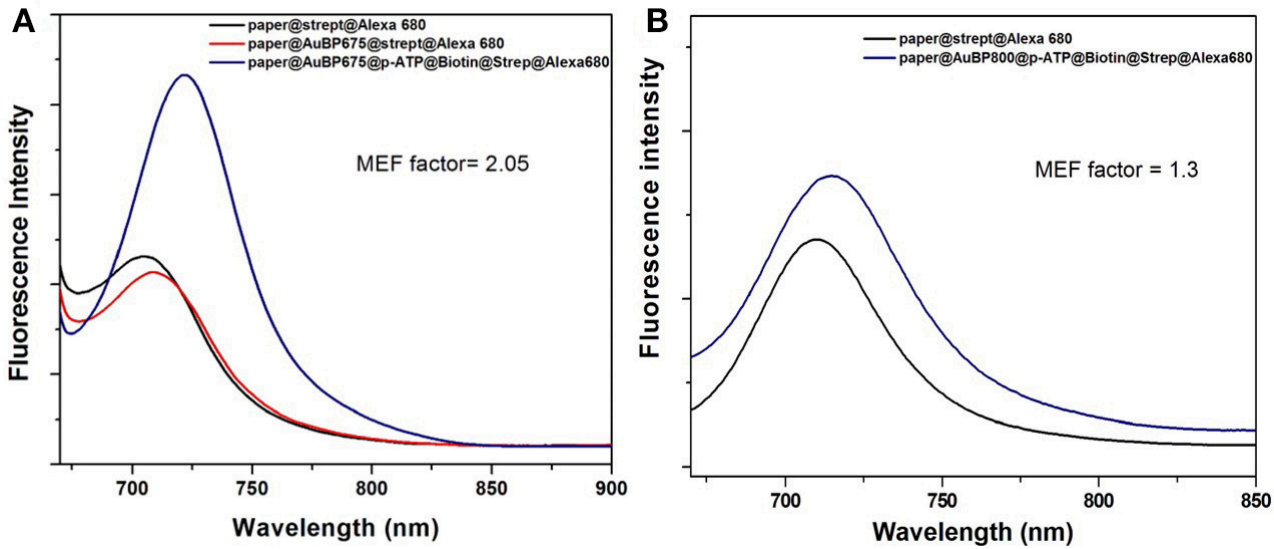
Emission spectra of the streptavidin@Alexa 680 complex onto the biotinylated plasmonic lines (blue spectra), namely paper@AuBP675 **(A)** and paper@AuBP800 **(B)** together with the emission spectra of the streptavidin@Alexa 680 (black spectra) on bare Whatman paper (near the plasmonic lines). Excitation wavelength at 675 nm.

Finally, we were interested in examining the effective MEF capability of our designed plasmonic nanoplatforms, by comparing the fluorescence of Alexa 680 fluorophore in the streptavidin@Alexa 680 complex immobilized onto the biotinylated plasmonic lines with its fluorescence emission on bare paper (near the plasmonic line, without immobilized AuBPs onto the paper) in the same experimental conditions. In fact, when the specific biotin-streptavidin interaction was realized, this binding acts as optimal spacer between AuBPs surface and Alexa 680 fluorophore, preventing the quenching process, the streptavidin@Alexa 680 on paper@AuBP675 (Figure 5A-blue spectra) exhibits up to a 2-fold fluorescence emission enhancement as compared to streptavidin@Alexa 680 onto the bare paper (Figure 5A-black spectra). It is worth mentioning, that the emission spectra of the captured streptavidin@Alexa 680 onto the biotinylated plasmonic lines were measured while employing a 675 nm excitation wavelength, which corresponds to the simultaneous excitation of Alexa 680 and paper@AuBP675 via the absorption band and the longitudinal LSPR.

All the fluorescence spectra were collected under identical excitation and experimental conditions and, for this reason, the average MEF factor can be determinate directly from the ratio between the two recorded emission intensities. As a result, Figure 5 shows that all emission spectra collected from streptavidin@Alexa 680 captured on plasmonic lines are enhanced relative to the emission spectra collected from streptavidin@Alexa 680 on bare paper (near plasmonic lines), but the resulting MEF factor is slightly different for different spectral overlapping between the longitudinal LSPR bands and the fluorophore absorption band. In fact, AuBPs are well-known to concentrate at their tips a locally enhanced-electromagnetic field, which is essential for the enhancement of the fluorescence of Alexa 680. Therefore, we admit that the MEF effect in our case is due to the increase of the radiative emission rate when AuBPs act as effective plasmonic nanoantenna and the increase of excitation rate when surface plasmon are resonantly excited.

## Conclusions

In summary, we developed a new flexible and tunable paper-based nanoplatform having different active plasmonic lines with modulated LSPR responses designed by a simple and inexpensive plasmonic calligraphy approach using a commercial pen filled with AuBPs as colloidal inks. The immobilization of the AuBPs on the cellulose fibers was successfully confirmed using SEM and HRTEM investigations. By simply retracing new plasmonic lines in the same position, we were able to obtain a versatile manipulation of the optical density of our nanoplatforms. Subsequently, the high potential of our designed paper-based platforms to be employed as efficient biosensors with multiplex capability was validated by detecting the specific biotin-streptavidin interaction as a “proof-of-concept.” With this reason, the p-ATB@Biotin complex was easily grafted on our plasmonic lines through strong Au-S interaction, representing not only the recognition element for the specific streptavidin protein detection from streptavidin@Alexa680 system, but also the spacer needed for ensuring an optimal metal-fluorophore distance necessary for further implementation as innovative MEF biosensing platforms. Therefore, the LSPR based sensing capabilities of the tunable plasmonic lines on paper were firstly proved by the successive 13–18 nm red-shifts of the longitudinal LSPR band after each binding step, such as the p-ATP@Biotin immobilization followed by the specific capture of streptavidin. Secondly, the molecular identification of biotin-streptavidin interaction was confirmed via ultrasensitive SERS detection by the monitorization of the p-ATP vibrational modes along with the amplification of the characteristic Raman bands of the protein residues. Finally, the MEF sensing abilities of the paper-based nanosensors were confirmed by monitoring the enhancement of the Alexa 680 emission using epifluorescence measurements. As a result, compared with the emission of streptavidin@Alexa 680 on bare paper a 2.05-fold fluorescence enhancement was recorded when both Alexa 680 fluorophore and paper@AuBPs675 were resonantly excited. In conclusion, considering the significant importance of the development of miniaturized POC diagnostics test, our inexpensive calligraphic paper-based plasmonic nanoplatform can represent an excellent candidate in the future for promoting multimodal detection of relevant biomarkers.

## Author Contributions

MF conceptualization. AC, LS, AMC, FO, and DM fabrication and validation of the plasmonic nanoplatforms. AV performed the SEM measurements. LR performed the HRTEM measurements. AC and LS writing-original draft preparation. MF final writing-review and editing. SA final review. All authors reviewed the manuscript.

### Conflict of Interest Statement

The authors declare that the research was conducted in the absence of any commercial or financial relationships that could be construed as a potential conflict of interest.

## References

[B1] Ashley M. J. Bourgeois M. R. Murthy R. R. Laramy C. R. Ross M. B. Naik R. R. (2018). Shape and size control of substrate-grown gold nanoparticles for surface-enhanced raman spectroscopy detection of chemical analytes. J. Phys. Chem. C 122, 2307–2314. 10.1021/acs.jpcc.7b11440

[B2] Aslan K. Lakowicz J. R. Geddes C. D. (2005). Plasmon light scattering in biology and medicine: new sensing approaches, visions and perspectives. Curr. Opin. Chem. Biol. 9, 538–544. 10.1016/j.cbpa.2005.08.021 16129649 PMC6816254

[B3] Azizi Samir M. A. S. Alloin F. Dufresne A. (2005). Review of recent research into cellulosic whiskers, their properties and their application in nanocomposite field. Biomacromolecules 6, 612–626. 10.1021/bm0493685 15762621

[B4] Campu A. Lerouge F. Chateau D. Chaput F. Baldeck P. Parola S.. (2018). Gold nanobipyramids performing as highly sensitive dual-modal optical immunosensors. Anal. Chem. 90, 8567–8575. 10.1021/acs.analchem.8b01689 29902917

[B5] Chateau D. Liotta A. Vadcard F. Navarro J. R. G. Chaput F. Lermé J.. (2015). From gold nanobipyramids to nanojavelins for a precise tuning of the plasmon resonance to the infrared wavelengths: experimental and theoretical aspects. Nanoscale 7, 1934–1943. 10.1039/c4nr06323f 25530122

[B6] Dalla Marta S. Novara C. Giorgis F. Bonifacio A. Sergo V. (2017). Optimization and characterization of paper-made surface enhanced raman scattering (SERS) substrates with Au and Ag NPs for quantitative analysis. Materials 10:1365. 10.3390/ma10121365 29182585 PMC5744300

[B7] Della Ventura B. Gelzo M. Battista E. Alabastri A. Schirato A. Castaldo G.. (2019). Biosensor for point-of-care analysis of immunoglobulins in urine by metal enhanced fluorescence from gold nanoparticles. ACS Appl. Mater. Interfaces. 11, 3753–3762. 10.1021/acsami.8b20501 30609355

[B8] Deng W. Goldys E. M. (2012). Plasmonic approach to enhanced fluorescence for applications in biotechnology and the life sciences. Langmuir 28, 10152–10163. 10.1021/la300332x 22568517

[B9] Galarreta B. C. Norton P. R. Lagugné-Labarthet F. (2011). SERS Detection of streptavidin/biotin monolayer assemblies^†^. Langmuir 27, 1494–1498. 10.1021/la1047497 21244074

[B10] Geddes C. D. Lakowicz J. R. (2002). Editorial: metal-enhanced fluorescence. J. Fluoresc. 12, 121–129. 10.1023/A:1016875709579

[B11] Hankus M. E. Tian L. Pellegrino P. M. Singamaneni S. (2012). Plasmonic paper as a highly efficient SERS substrate, in Proceedings Volume 8358, Chemical, Biological, Radiological, Nuclear, and Explosives (CBRNE) Sensing XIII (Baltimore, MD). 10.1117/12.919415

[B12] Jiang Q. Chandar Y. J. Cao S. Kharasch E. D. Singamaneni S. Morrissey J. J. (2017). Rapid, point-of-care, paper-based plasmonic biosensor for zika virus diagnosis. Adv. Biosyst. 1:1700096 10.1002/adbi.201700096 32646188

[B13] Jiang X. Ahmed M. Deng Z. Narain R. (2009). biotinylated glyco-functionalized quantum dots: synthesis, characterization, and cytotoxicity studies. Bioconjug. Chem. 20, 994–1001. 10.1021/bc800566f 19402705

[B14] Kim W. Lee S. H. Kim J. H. Ahn Y. J. Kim Y.-H. Yu J. S.. (2018). Paper-based surface-enhanced raman spectroscopy for diagnosing prenatal diseases in women. ACS Nano 12, 7100–7108. 10.1021/acsnano.8b02917 29920065

[B15] Lee C. H. Hankus M. E. Tian L. Pellegrino P. M. Singamaneni S. (2011). Highly sensitive surface enhanced raman scattering substrates based on filter paper loaded with plasmonic nanostructures. Anal. Chem. 83, 8953–8958. 10.1021/ac2016882 22017379

[B16] Li J. Wang X. Huo D. Hou C. Fa H. Yang M. (2017). Colorimetric measurement of Fe3+ using a functional paper-based sensor based on catalytic oxidation of gold nanoparticles. Sens. Actuators B Chem. 242, 1265–1271. 10.1016/j.snb.2016.09.039

[B17] Li Q. Zhuo X. Li S. Ruan Q. Xu Q.-H. Wang J. (2015). Production of monodisperse gold nanobipyramids with number percentages approaching 100% and evaluation of their plasmonic properties. Adv. Opt. Mater. 3, 801–812. 10.1002/adom.201400505

[B18] Li S. Zhang L. Jiang Y. Zhu S. Lv X. Duan Z.. (2017). In-site encapsulating gold “nanowires” into hemin-coupled protein scaffolds through biomimetic assembly towards the nanocomposites with strong catalysis, electrocatalysis, and fluorescence properties. Nanoscale 9, 16005–16011. 10.1039/C7NR04945E 29022633

[B19] Liana D. D. Raguse B. Gooding J. J. Chow E. (2012). Recent advances in paper-based sensors. Sensors 12, 11505–11526. 10.3390/s120911505 23112667 PMC3478794

[B20] Liu J. Li S. Bhethanabotla V. R. (2018). Integrating metal-enhanced fluorescence and surface acoustic waves for sensitive and rapid quantification of cancer biomarkers from real matrices. ACS Sens. 3, 222–229. 10.1021/acssensors.7b00876 29284267

[B21] Liu M. Guyot-Sionnest P. (2005). Mechanism of silver(I)-assisted growth of gold nanorods and bipyramids. J. Phys. Chem. B 109, 22192–22200. 10.1021/jp054808n 16853888

[B22] Mayer K. M. Hafner J. H. (2011). Localized surface plasmon resonance sensors. Chem. Rev. 111, 3828–3857. 10.1021/cr100313v 21648956

[B23] Navarro J. R. G. Manchon D. Lerouge F. Cottancin E. Lermé J. Christophe B.. (2012). Synthesis, electron tomography and single-particle optical response of twisted gold nano-bipyramids. Nanotechnology 23:145707. 10.1088/0957-4484/23/14/145707 22433232

[B24] Ngo Y. H. Then W. L. Shen W. Garnier G. (2013). Gold nanoparticles paper as a SERS bio-diagnostic platform. J. Colloid Interface Sci. 409, 59–65. 10.1016/j.jcis.2013.07.051 23978290

[B25] Nooney R. Clifford A. LeGuevel X. Stranik O. McDonagh C. MacCraith B. D. (2010). Enhancing the analytical performance of immunoassays that employ metal-enhanced fluorescence. Anal. Bioanal. Chem. 396, 1127–1134. 10.1007/s00216-009-3357-9 20012901

[B26] Oliveira M. J. Quaresma P. Peixoto de Almeida M. Araújo A. Pereira E. Fortunato E.. (2017). Office paper decorated with silver nanostars - an alternative cost effective platform for trace analyte detection by SERS. Sci. Rep. 7:2480. 10.1038/s41598-017-02484-8 28559536 PMC5449394

[B27] Park M. Hwang C. S. H. Jeong K.-H. (2018). Nanoplasmonic alloy of Au/Ag nanocomposites on paper substrate for biosensing applications. ACS Appl. Mater. Interfaces 10, 290–295. 10.1021/acsami.7b16182 29220574

[B28] Pompa P. P. Martiradonna L. Torre A. D. Sala F. D. Manna L. De Vittorio M.. (2006). Metal-enhanced fluorescence of colloidal nanocrystals with nanoscale control. Nat. Nanotechnol. 1, 126–130. 10.1038/nnano.2006.93 18654164

[B29] Rao W. Li Q. Wang Y. Li T. Wu L. (2015). Comparison of photoluminescence quantum yield of single gold nanobipyramids and gold nanorods. ACS Nano 9, 2783–2791. 10.1021/nn506689b 25665929

[B30] Reguera J. Langer J. Aberasturi D. J., de Liz-Marzán L. M. (2017). Anisotropic metal nanoparticles for surface enhanced Raman scattering. Chem. Soc. Rev. 46, 3866–3885. 10.1039/C7CS00158D 28447698

[B31] Rye J.-M. Bonnet C. Lerouge F. Pellarin M. Lermé J. Parola S.. (2018). Single gold bipyramids on a silanized substrate as robust plasmonic sensors for liquid environments. Nanoscale 10, 16094–16101. 10.1039/C8NR03400A 30109878

[B32] Si Y. Sun Z. Zhang N. Qi W. Li S. Chen L.. (2014). Ultrasensitive Electroanalysis of low-level free microRNAs in blood by maximum signal amplification of catalytic silver deposition using alkaline phosphatase-incorporated gold nanoclusters. Anal. Chem. 86, 10406–10414. 10.1021/ac5028885 25242013

[B33] Stewart M. E. Anderton C. R. Thompson L. B. Maria J. Gray S. K. Rogers J. A.. (2008). Nanostructured plasmonic sensors. Chem. Rev. 108, 494–521. 10.1021/cr068126n 18229956

[B34] Tian L. Jiang Q. Liu K.-K. Luan J. Naik R. R. Singamaneni S. (2016). Bacterial nanocellulose-based flexible surface enhanced raman scattering substrate. Adv. Mater. Interfaces 3:1600214 10.1002/admi.201600214

[B35] Tian L. Tadepalli S. Hyun Park S. Liu K.-K. Morrissey J. J. Kharasch E. D.. (2014). Bioplasmonic calligraphy for multiplexed label-free biodetection. Biosens. Bioelectron. 59, 208–215. 10.1016/j.bios.2014.03.043 24727607 PMC4044868

[B36] Zhang L. Fan C. Liu M. Liu F. Bian S. Du S. (2018). Biominerized gold-Hemin@MOF composites with peroxidase-like and gold catalysis activities: a high-throughput colorimetric immunoassay for alpha-fetoprotein in blood by ELISA and gold-catalytic silver staining. Sens. Actuators B Chem. 266, 543–552. 10.1016/j.snb.2018.03.153

[B37] Zhang N. Si Y. Sun Z. Chen L. Li R. Qiao Y.. (2014). Rapid, selective, and ultrasensitive fluorimetric analysis of mercury and copper levels in blood using bimetallic gold–silver nanoclusters with “Silver Effect”-enhanced red fluorescence. Anal. Chem. 86, 11714–11721. 10.1021/ac503102g 25350497

[B38] Zhang S. Xiong R. Mahmoud M. A. Quigley E. N. Chang H. El-Sayed M.. (2018). Dual-excitation nanocellulose plasmonic membranes for molecular and cellular SERS detection. ACS Appl. Mater. Interfaces 10, 18380–18389. 10.1021/acsami.8b04817 29737825

[B39] Zhu X. Zhuo X. Li Q. Yang Z. Wang J. (2016). Gold nanobipyramid-supported silver nanostructures with narrow plasmon linewidths and improved chemical stability. Adv. Funct. Mater. 26, 341–352. 10.1002/adfm.201503670

[B40] Zong S. Wang Z. Yang J. Cui Y. (2011). Intracellular pH sensing using *p*-aminothiophenol functionalized gold nanorods with low cytotoxicity. Anal. Chem. 83, 4178–4183. 10.1021/ac200467z 21513305

