# Supplementary material for: Multimodal Biosensing on Paper-Based Platform Fabricated by Plasmonic Calligraphy Using Gold Nanobypiramids Ink
Source: Front Chem. 2019 Feb 8;7:55. doi: 10.3389/fchem.2019.00055 (PMC6375850; doi:10.3389/fchem.2019.00055)
Supplement: Supplementary file 1 [file Data_Sheet_1.PDF]

Supplementary Material

Multimodal biosensing on paper-based platform fabricated by plasmonic calligraphy using gold nanobypiramids ink

Andreea Campu[1], Laurentiu Susu[1], Filip Orzan[1], Dana Maniu[2], Ana Maria Craciun[1], Adriana Vulpoi[3], Lucian Roiban[4], Monica Focsan[1*], Simion Astilean[1,2*]

[1] Nanobiophotonics and Laser Microspectroscopy Center, Interdisciplinary Research Institute on Bio-Nano-Sciences, Babes-Bolyai University, Treboniu Laurean No. 42, Cluj-Napoca 400271, Romania

[2] Biomolecular Physics Department, Faculty of Physics, Babes-Bolyai University, M Kogalniceanu No. 1, Cluj-Napoca 400084, Romania

[3] Nanostructured Materials and Bio-Nano-Interfaces Center, Interdisciplinary Research Institute on Bio-Nano-Sciences, Babes-Bolyai University, Treboniu Laurian Str. 42, Cluj-Napoca 400271, Romania

[4] Univ Lyon, INSA-Lyon, Université Claude Bernard Lyon 1, MATEIS, UMR 5510, CNRS, 69621 Villeurbanne Cedex, France

*** Correspondence:**
Corresponding Authors

monica.iosin@phys.ubbcluj.ro
simion.astilean@phys.ubbcluj.ro

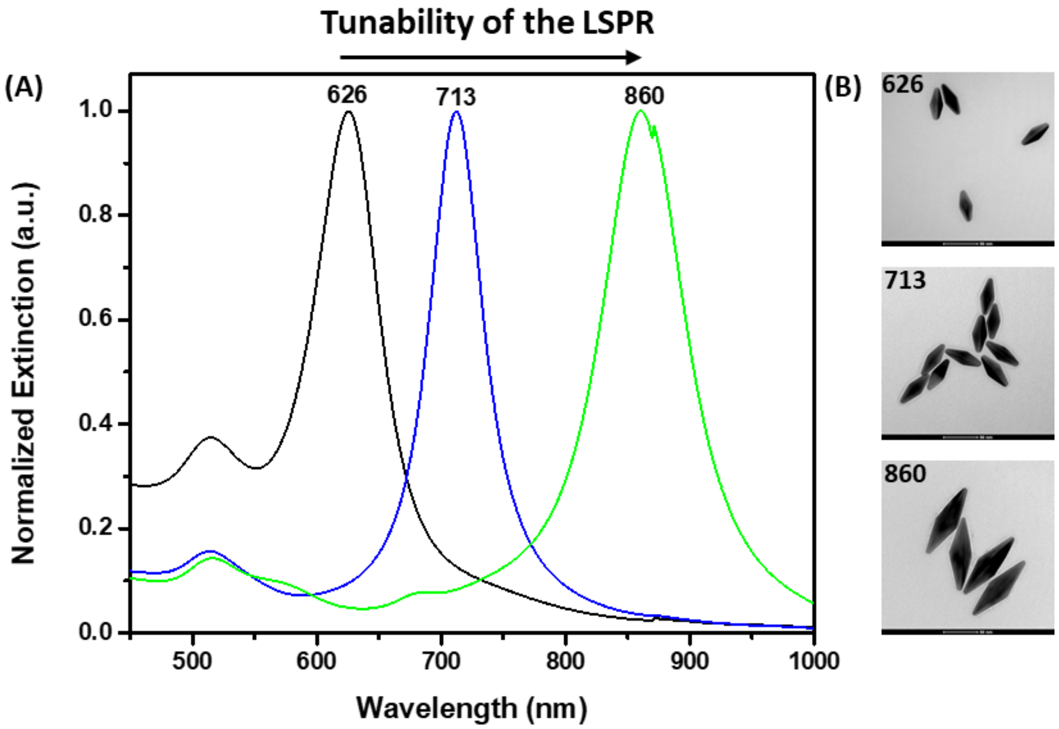

Supplementary Figure 1. Normalized extinction spectra of three different synthesized colloidal AuBPs **(A)** and their corresponding TEM images **(B)**.

LSPR response	AuBPs 626	AuBPs 713	AuBPs 860
TEM	length = 50 ± 3 nm<br>width = 21 ± 1 nm	length = 58 ± 2 nm<br>width= 22 ± 1 nm	length = 115 ± 3 nm<br>width = 34 ± 1 nm
Hydrodynamic diameter	65 nm	74 nm	126 nm
Zeta Potential	27 mV	27 mV	28 mV

Supplementary Table 1. The recorded maximum position of the longitudinal LSPR band together with the corresponding hydrodynamic diameter and zeta potential data for each synthesized AuBPs in aqueous solution.

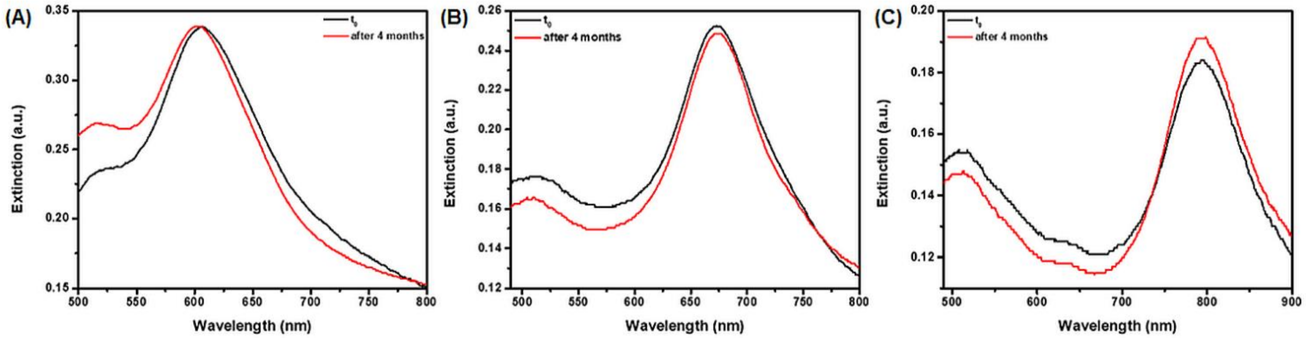

Supplementary Figure 2. LSPR responses of the as-designed paper-based plasmonic nanoplatforms with tunable plasmonic response at paper@AuBP 618 (A), paper@AuBP 675 (B) and paper@AuBP 800 (C) immediately after their fabrication (black spectra) and after 4 months (red spectra).

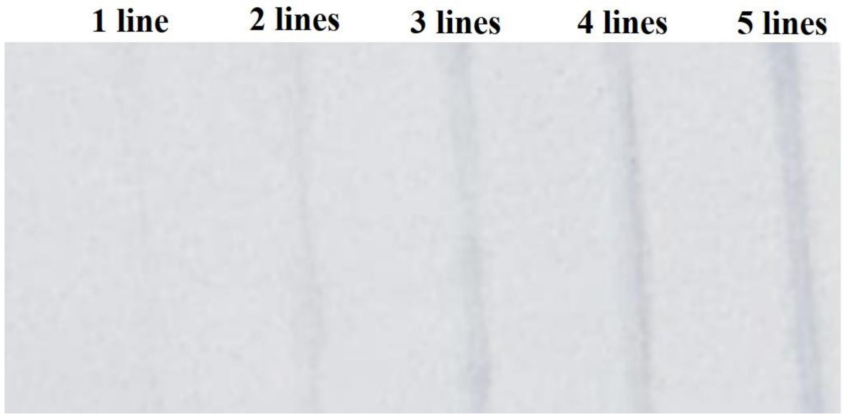

Supplementary Figure 3. A photograph representing the writing process directly on Whatman paper using a commercial pen filled with concentrated colloidal AuBPs, employed herein as plasmonic ink, to draw spatially isolated lines. The number of plasmonic lines increase from left to right.

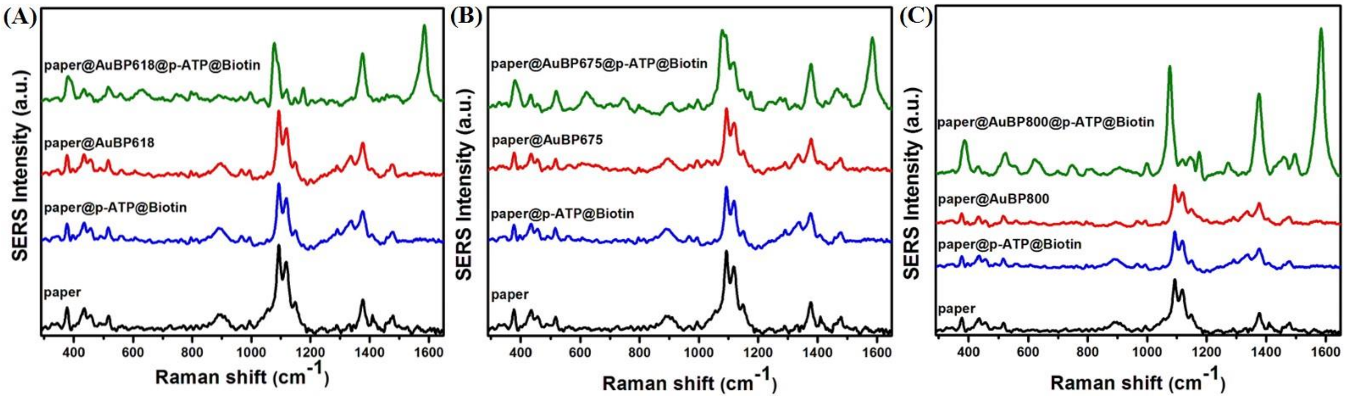

Supplementary Figure 4. The Raman spectra of bare Whatman paper (black spectra), p-ATP@Biotin complex on bare Whatman paper (blue spectra) and paper@AuBPs with different optical responses (red spectra) and SERS spectra recorded after the biotinylating step (green spectra) on the as-designed plasmonic lines on paper, i.e paper@AuBP 618 (A), paper@AuBP 675 (B) and paper@AuBP 800 (C), respectively. Excitation: 785 nm laser line

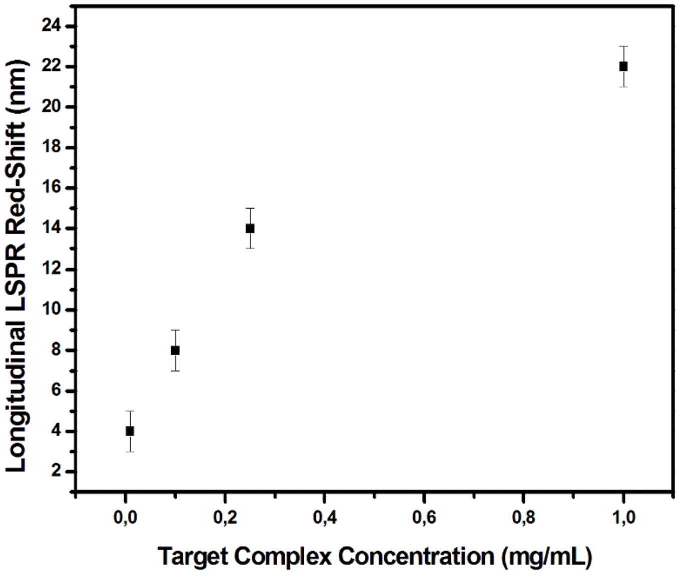

Supplementary Figure 5. LOD determined *via* LSPR by measuring the LSPR shift of the p-ATP@Biotin grafted on the paper@AuBP 675 nanoplatform with different concentrations of target complex concentrations.
